# Supplementary material for: Intelligent Device for Harvesting the Vibration Energy of the Automobile Exhaust with a Piezoelectric Generator
Source: Micromachines (Basel). 2023 Feb 20;14(2):491. doi: 10.3390/mi14020491 (PMC9960593; doi:10.3390/mi14020491)
Supplement: Supplementary file 1 [file micromachines-14-00491-s001.zip › micromachines-2221829 supplementary.pdf]

Supporting Information

Intelligent Device for Harvesting the Vibration Energy of the Automobile Exhaust with a Piezoelectric Generator

Jie Huang*[1], Cheng Xu[2], Nan Ma[3], Qinghui Zhou[4], Zhaohua Ji[1], Chunxia Jia[1], Shan Xiao[1], Peng Wang[1]

[1]*Beijing Information Technology College, Beijing, China*

[2]*Beijing Key Laboratory of Information Service Engineering, Beijing Union University, Beijing, China*

[3]*Beijing University of Technology, Beijing, China*

[4]*Beijing University of Civil Engineering and Architecture, Beijing, China*

*Corresponding authors' e-mail addresses: huangj-n@bitc.edu.cn.

This is the mounting platform used to verify the performance of a single PZT. The mounting platform is divided into stator and rotor (Figure S1a, b). The stator is fixed on the angle iron and the rotor is fixed on the linear motor. The piezoelectric sheet and the paddle are respectively fixed on the stator and the mover. The linear motor tests the basic performance of PZT by simulating the vibration environment. The assembly diagram is shown in Figure S1c.

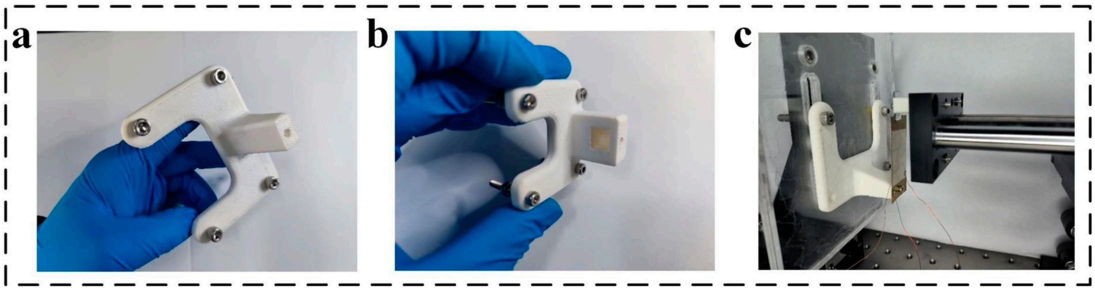

Figure S1. Physical structure of installation platform: (a) stator, (b) rotor, (c) assembly drawing.

The current and power performance of different specifications of PZT are shown in Figure S2. The current increases as the excitation stroke increases. The maximum power of PZT-2 is 151 μW when the excitation is 5 mm, and the power of PZT-1 and PZT-3 are 37 and 43 μW respectively.

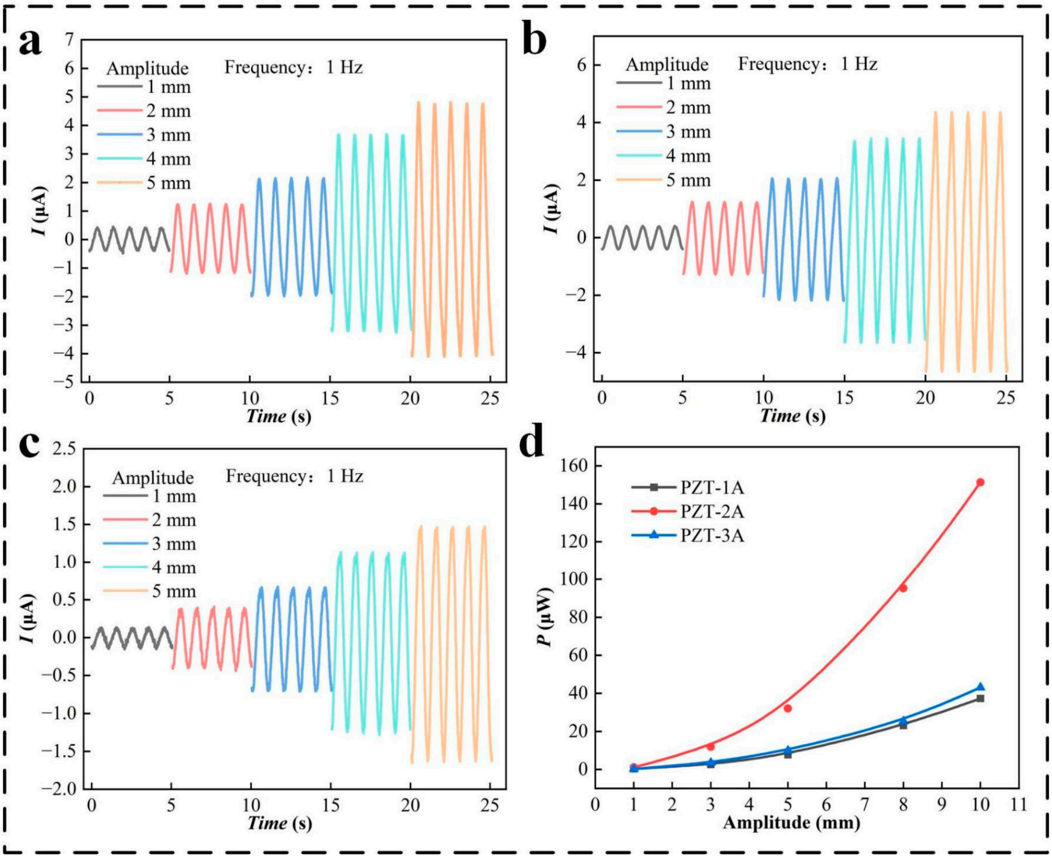

Figure S2. Output performance of the PZT units having different specifications: (a-c) current of PZT units of different specifications for a stroke of the motor of 1, 2, 3, 4, and 5 mm, (d) power of different PZT for a stroke of the motor of 1, 2, 3, 4, and 5 mm.

Figure S3a shows the physical picture of the high-frequency vibration platform. PZT is installed on the base-A and the paddle is installed on the base-B. Base-B is driven by a vibration motor to generate vibration, and the voltage signal is tested by an oscilloscope. Figure S3b and c is a schematic diagram of series and parallel circuits.

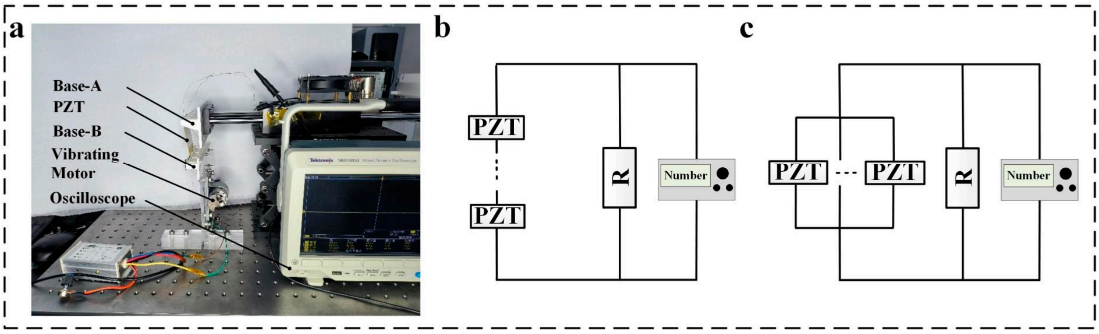

Figure S3. (a) Physical structure of high-frequency vibration test bench. (b-c) Circuit diagram of PZT in series and parallel.

Figure S4 a-c shows that PZT releases different current according to the magnitude of random amplitude. Figure S4d shows the power curve of PZT of different specifications in high-frequency vibration experiment, and it can be seen that the power curve of PZT-2 is relatively high.

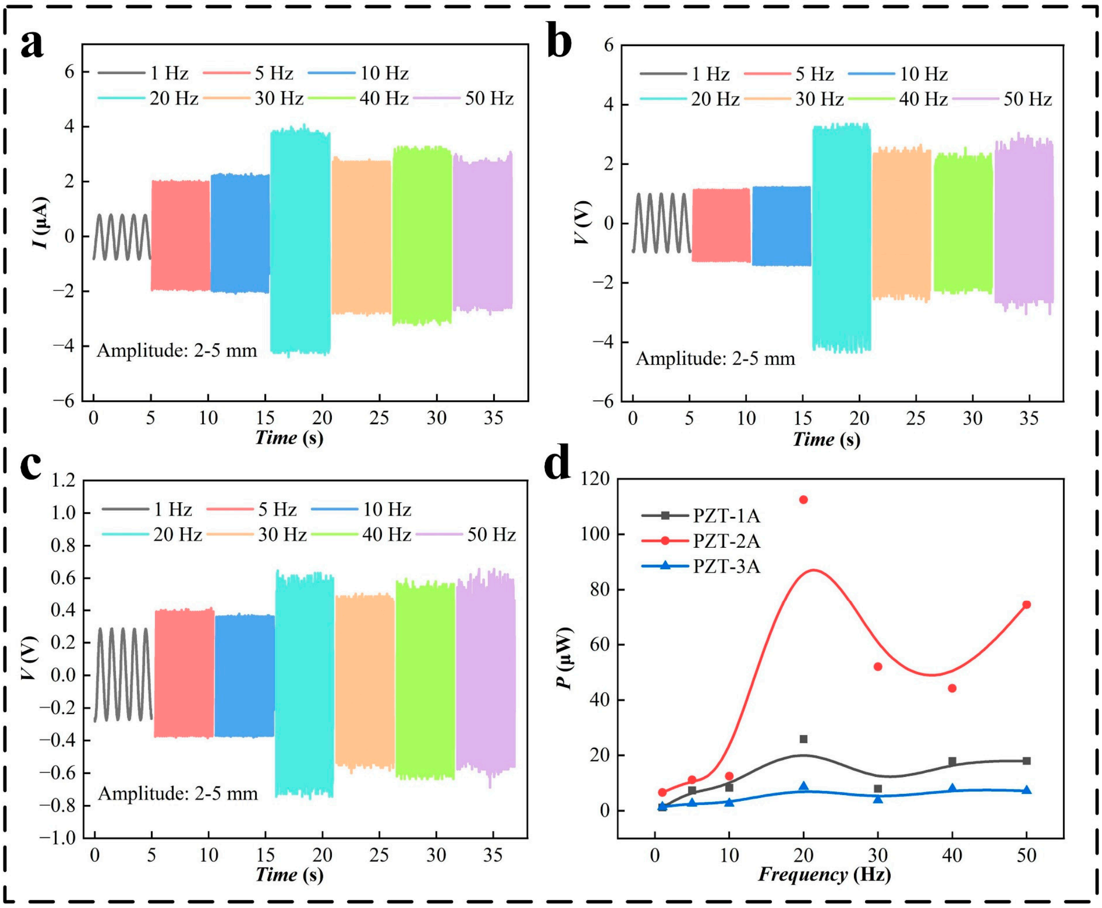

Figure S4. Output performance of PZT of different specifications: (a-c) the current and power of different specifications of PZT. (d) power of different PZT at different frequencies.

Figure S5 shows the voltage performance of each unit of PZT 1-4.

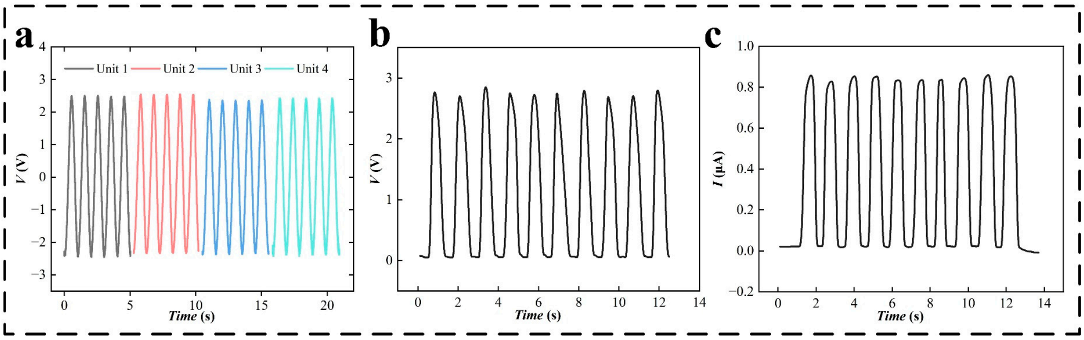

Figure S5. (a) Voltage of the PZT 1-4. (b-c) rectified voltage and current.
